# *Azadirachta indica* ethanolic extract protects neurons from apoptosis and mitigates brain swelling in experimental cerebral malaria

**DOI:** 10.1186/1475-2875-12-298

**Published:** 2013-08-29

**Authors:** Selma Bedri, Eltahir A Khalil, Sami A Khalid, Mohammad A Alzohairy, Abdlmarouf Mohieldein, Yousef H Aldebasi, Paul Faustin Seke Etet, Mohammed Farahna

**Affiliations:** 1 Department of Clinical Pathology and Immunology, Institute of Endemic Diseases, University of Khartoum, Khartoum, Sudan; 2 Department of Pharmacognosy, Faculty of Pharmacy, University of Khartoum, Khartoum, Sudan; 3 Department of Laboratory Medicine, College of Applied Medical Sciences, Qassim University, Buraydah 51452, Saudi Arabia; 4 Department of Optometry, College of Applied Medical Sciences, Qassim University, Buraydah 51452, Saudi Arabia; 5 Department of Basic Health Sciences, College of Applied Medical Sciences, Qassim University, Buraydah 51452, Saudi Arabia

**Keywords:** Cerebral malaria, *Azadirachta indica* extract, Pyramidal neurons, Neuroinflammation, Brain oedema

## Abstract

**Background:**

Cerebral malaria is a rapidly developing encephalopathy caused by the apicomplexan parasite *Plasmodium falciparum*. Drugs currently in use are associated with poor outcome in an increasing number of cases and new drugs are urgently needed. The potential of the medicinal plant *Azadirachta indica* (Neem) for the treatment of experimental cerebral malaria was evaluated in mice.

**Methods:**

Experimental cerebral malaria was induced in mice by infection with *Plasmodium berghei* ANKA. Infected mice were administered with *Azadirachta indica* ethanolic extract at doses of 300, 500, or 1000 mg/kg intraperitoneally (i.p.) in experimental groups, or with the anti-malarial drugs chloroquine (12 mg/kg, i.p.) or artemether (1.6 mg/kg, i.p.), in the positive control groups. Treatment was initiated at the onset of signs of brain involvement and pursued for five days on a daily basis. Mice brains were dissected out and processed for the study of the effects of the extract on pyramidal cells’ fate and on markers of neuroinflammation and apoptosis, in the medial temporal lobe.

**Results:**

*Azadirachta indica* ethanolic extract mitigated neuroinflammation, decreased the severity of brain oedema, and protected pyramidal neurons from apoptosis, particularly at the highest dose used, comparable to chloroquine and artemether.

**Conclusions:**

The present findings suggest that *Azadirachta indica* ethanolic extract has protective effects on neuronal populations in the inflamed central nervous system, and justify at least in part its use in African and Asian folk medicine and practices.

## Background

Cerebral malaria is a rapidly developing encephalopathy that represents the most severe neurological complication of infection with *Plasmodium falciparum*. Cerebral malaria mainly occurs in resource-poor tropical countries, and mostly affects children [1,2]. Surviving patients display neurologic and cognitive deficits, including consciousness impairment, cerebellar ataxia, seizures, and coma, making cerebral malaria a major cause of childhood neurodisability in endemic areas [3-6]. The pathogenesis of neuro-cognitive sequelae is poorly understood.

Experimental models of cerebral malaria have been providing new mechanistic insights that will help develop efficient neuro-protective interventions. The non-human pathogenic parasite *Plasmodium berghei* has been used to induce experimental cerebral malaria in mice that has some features comparable to the human disease [7-9]. Experimental evidence suggests that the vast array of pro-inflammatory molecules released by the host to fight the infection and alterations due to parasite sequestration in the microvessels cause leakage of plasma into the parenchyma resulting in brain oedema, and may contribute to the subsequent blood–brain barrier breakdown [10,11]. Brain parenchyma infiltration by danger or pathogen-associated molecules would trigger a sustained detrimental neuroinflammatory response leading to the recruitment of circulating immune cells. The latter cells would induce death through cell-mediated apoptosis in sensible neuronal populations, by triggering pathways such as caspase 3 and Fas-Fas ligand [12-14]. Previous studies and reports have shown that such inflammation-triggered apoptosis can induce cerebellar ataxia when affecting Purkinje cells in the cerebellum [15-17]. Similarly, apoptosis affecting cortical neurons may explain many other neurologic deficits observed in experimental cerebral malaria, such as cognitive impairment or seizures [18-21]. Overall, these findings corroborate the alterations reported in humans [9,14,22,23]. New effective drugs are urgently needed for cerebral malaria treatment due to the development of parasite chemoresistance, and to improve current drugs’ poor treatment outcome [24-26].

*Azadirachta indica* is a plant commonly used in the African and Asian folk medicine and practices [27-31]. Anti-malarial effects of *A. indica* extracts have been demonstrated [32-36], as well as the neuro-protective effects on Purkinje cells of *P. berghei-*infected mice successfully mitigating cerebellum malaria [16]. The present study addresses the potential of *A. indica* extract for the protection of cortical and sub-cortical pyramidal neurons of *P. berghei*-infected mice, considering the implications for the treatment of cerebral malaria-associated cortical deficits.

## Methods

### Experimental procedure

Thirty five mice, divided into seven groups (five animals per group), were used in this study. These groups were: *i*) a disease control group made of untreated *P. berghei-*infected mice; *ii*) two positive control groups made of *P. berghei-*infected mice treated with the commercially available anti-malarial drugs chloroquine (12 mg/kg in distilled water, i.p.) and artemether (1.6 mg/kg in olive oil, i.p.); *iii*) three experimental groups made of *P. berghei-*infected mice treated with *A. indica* ethanolic extract at doses 300, 500, or 1000 mg/kg (i.p.); *iv*) and a group of uninfected healthy animals. Parasitaemia, body weight, core body temperature, and signs of brain involvement were monitored. Treatment was started when the number of parasitized red blood cells was >5% (severe malaria indicator) and was associated with signs of brain involvement. Treatment was pursued for five days on a daily basis. Mice either died spontaneously or were sacrificed by ether inhalation-induced deep anesthesia once signs of disease terminal stage (hypothermia, ptosis, and convulsion) [37], were observed. Uninfected mice were sacrificed concomitantly with the last animals to check for disease terminal stage signs. For all animals, brains were dissected out and processed for histopathological analysis.

### Animals and infection

Male Swiss albino mice (weighing 26.5 ± 3.0 g, aged 6 ± 1 week) were donated by Sudan National Health Laboratory (Khartoum, Sudan). Mice were maintained at a 12:12 light/dark cycle and were given ad libitum access to food (standard diet) and water. For infection, mice were administered (i.p.) with a load of 10 × 10^6^ parasitized red blood cells containing *P. berghei* ANKA. The parasitaemia was checked by blood smear Giemsa-staining. All procedures received approval from the Ethical Committee of the Institute of Endemic Diseases of the University of Khartoum and animals were handled according to institutional guidelines. *Plasmodium berghei* ANKA blood-stage cryostabilates were kindly donated by Dr. C.R. Pillai (National Institute of Malaria, New Delhi, India).

### Preparation of the extract

Fresh leaves of *A. indica* were collected in Khartoum region, where they are commonly used in folk medicine to treat malaria [33], and shade-dried. Dry leaves were grinded, and 1500 g of powder were macerated in 15 L of ethanol for three consecutive days at room temperature. A 1 mg/mL stock solution was obtained by filtration and boiling of the supernatant, and was stored at 4°C until use. Three doses of *A. indica*, i.e. 300, 500, and 1,000 mg/kg, were administered to the respective experimental groups once a day for five days. These regimens were chosen in accordance with previous pharmacological studies in malaria models [16,28].

### Pyramidal neuron density and brain oedema

Brains dissected out were fixed in 10% neutral buffered formaldehyde, then paraffin-embedded and cut sagittally (section thickness: 5 μm). Sections were deparaffinized in xylene, rehydrated, and processed for haematoxylin-eosin (H&E) staining. The pyramidal neuron volumetric density, i.e. the proportion of brain subfield that is occupied by pyramidal neuronal cell bodies, was determined as described by Highley and colleagues [38]. Pyramidal neurons were counted on five consecutive sections, in the medial temporal lobe, using a light microscope with a fixed 1 cm grid eyepiece under 40× objective. Brain oedema was assessed by giving a severity score integrating histopathological parameters, such as the importance (how big) and frequency (how numerous) of fluid built up in the brain parenchyma, on the H&E stained sections [39,40].

### Immunohistochemistry

The sections processed for immunohistochemical labeling were deparaffinized in xylene, rehydrated, and endogenous peroxidase activity was extinguished. Sections were then pre-incubated in normal serum buffer solution (Diagnostic BioSystems, Serpentine, CA, USA), and incubated for 3 h in either rabbit anti-caspase-3, anti-FAS, anti-FAS ligand, anti-tumor necrosis factor (TNF), or anti-nitric oxide synthase (NOS) primary antibody buffer solution (dilution factor 1:40, Lica Biosystem Newcastle Ltd, UK), followed by a biotinylated secondary antibody and streptavidin-conjugated horseradish peroxidase (Vision Biosystems Novocastra, Novocastra Laboratories Ltd., Newcastle, UK) prepared according to the kit instructions. The sections were incubated with 3,3′-diaminobenzidine hydrochloride (DAB) chromogen substrate (Vision Biosystems Novocastra) according to the manufacturer’s instructions, and counterstained with H&E. P value < .05 was considered significant. Thorough washes between steps were performed using immune wash buffer (Vision Biosystems Novocastra). Sections were dehydrated through a graded ethanol series, cleared in xylene, and covered with a thin glass coverslip. The fraction of pyramidal neurons expressing each of the studied markers, i.e. the markers of apoptosis caspase 3, Fas, and Fas ligand, and the markers of neuroinflammation-triggered apoptosis TNF and NOS, was determined using a light microscope under 40× objective.

### Statistical analysis

Data obtained from infected treated or uninfected groups were compared to those obtained from the infected untreated group using one-way ANOVA followed by LSD test. Differences with a p value < .05 were considered statistically significant. All data were expressed as a percentage of the value obtained in the infected untreated group. Data are presented as mean ± SEM (n = 5).

## Results

### General observations

The early signs of disease started five days after infection in the majority of *P. berghei-*infected animals, i.e. in the disease control group and in animals treated with chloroquine, artemether, or one of the three doses of *A. indica* extract. Cerebral malaria signs appeared at day 6 ± 1 post-infection. The most recurrent ones were a ruffled fur, locomotor impairments, and sickness behavior (anorexia, cachexia, fever) associated with body weight loss. Animals treated with *A. indica*, chloroquine or artemether did not displayed fever (as evaluated by increases in core body temperature), and from dose 500 mg/kg of extract locomotor disturbances and body weight loss were improved. Parasitaemia was decreased in infected mice following treatment with chloroquine or arthemether (from 6.1 ± 1.8% to 1.2 ± 0.4%, P < 0.05), but no significant change was observed in *A. indica*-treated animals, as previously reported [16].

*Azadirachta indica* extract did not protected infected mice from death, unlike chloroquine and artemether, although the highest doses slightly (not significantly) delayed death time. Infected untreated mice spontaneously died at day 9 ± 1 post-infection, *A. indica-*treated mice treated with the highest doses started to die at day 11, whereas no animal died in groups treated with chloroquine and artemether during the time of observation. However, about all the survivors in groups treated with the extract were sacrificed two days after (day 13 post-infection) as terminal signs of cerebral malaria, i.e. hypothermia, ptosis, ataxia, and convulsion, were observed. All other animals, including those treated with chloroquine or artemether as well as uninfected ones were also sacrificed then.

### Extract effect on pyramidal neuron density

**Figure 1:**
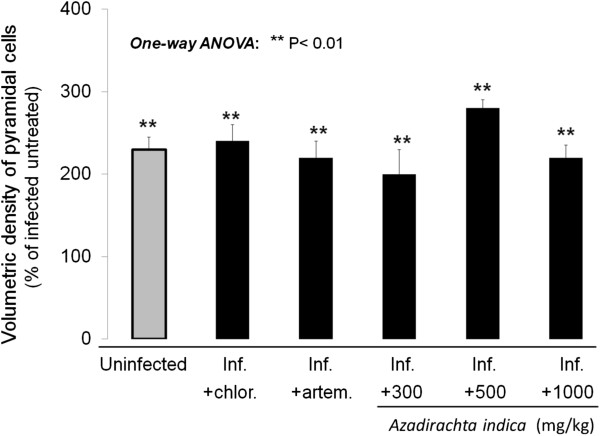
**Pyramidal cell density.** Effect of Azadirachta indica on the volumetric density of pyramidal neurons in Plasmodium berghei-infected (inf.) mice. Note the increase in volumetric density induced by treatment with chloroquine (chlor.), artemether (artem.), and all doses of A. indica extract used. Data are mean ± SEM.

The effect of *A. indica* on *P. berghei*-infected mice pyramidal neuron volumetric density is shown in Figure 1. The volumetric density of pyramidal neurons of mice treated with *A. indica* extract was significantly higher at all doses, in comparison to the infected untreated group (P < 0.01), and was comparable (non-significantly different [41]) to the values observed in the infected groups treated with chloroquine or artemether, and to uninfected group values, which were also significantly different from infected untreated group values (P < 0.01 for all three groups).

### Extract effect on caspase 3 expression

**Figure 2:**
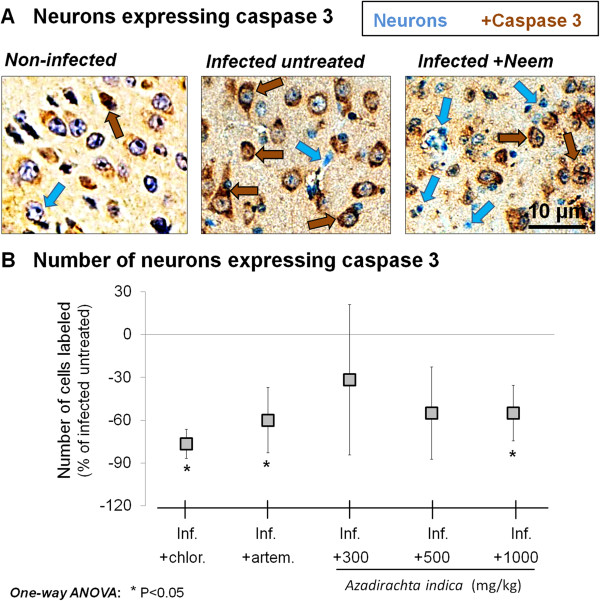
**Caspase 3 expression. A**. Micrographs showing the expression of caspase 3 in pyramidal neurons of representative examples of (from left): an uninfected mouse, an untreated Plasmodium berghei-infected mouse (inf.), and a P. berghei-infected mouse treated with Azadirachta indica crude extract. **B**. Effect of **A**. indica on caspase 3 expression in pyramidal neurons of P. berghei-infected mice. Note the significant decrease following treatment with chloroquine (chlor.), artemether (artem.), and **A**. indica extract (dose 1,000 mg/kg). Data are mean ± SEM.

The effect of *A. indica* on the expression of caspase 3 in pyramidal neurons of *P. berghei*-infected mice is shown in Figure 2. Pyramidal neurons of mice treated with *A. indica* extract displayed a significant decrease in caspase 3 expression at the highest dose, in comparison to the infected untreated group (P < 0.05), comparable to the decreases induced by chloroquine (P < 0.05 *vs*. infected untreated group) or artemether (P < 0.05 *vs*. infected untreated group).

### Extract effect on Fas and Fas ligand expression

**Figure 3:**
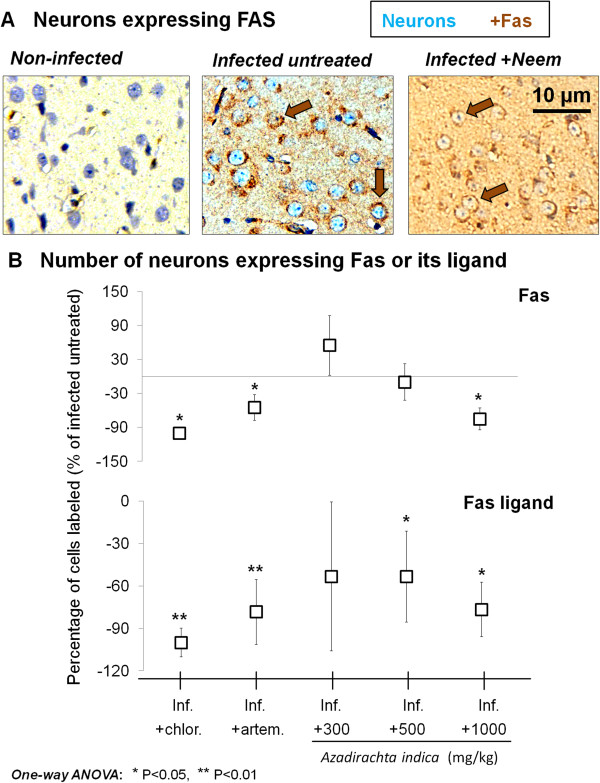
**Fas and Fas ligand expression. A**. Micrographs showing the expression of Fas in pyramidal neurons of representative examples of (from left): an uninfected mouse, an untreated Plasmodium berghei-infected mouse (inf.), and a P. berghei-infected mouse treated with Azadirachta indica crude extract. **B**. Effect of **A**. indica on the expression of Fas (upper) and Fas ligand (lower) in pyramidal neurons of P. berghei-infected mice. Note the significant decrease in expression of both molecules following treatment with chloroquine (chlor.), artemether (artem.), and **A**. indica extract (more marked at dose 1,000 mg/kg). Data are mean ± SEM.

The effect of *A. indica* on Fas and Fas ligand expression in *P. berghei*-infected mice pyramidal neurons is shown in Figure 3. Pyramidal neurons of mice treated with *A. indica* extract displayed a significant decrease in Fas expression at the highest dose, in comparison to the infected untreated group (P < 0.05) and a decrease in Fas ligand expression at doses 500 and 1000 mg/kg (P < 0.05 *vs*. infected untreated group). The significant decreases observed were comparable to those induced by chloroquine (P < 0.05 for Fas and P < 0.01 for Fas ligand *vs*. infected untreated group) or artemether (P < 0.05 for Fas and P < 0.01 for Fas ligand *vs*. infected untreated group).

### Extract effect on TNF expression

**Figure 4:**
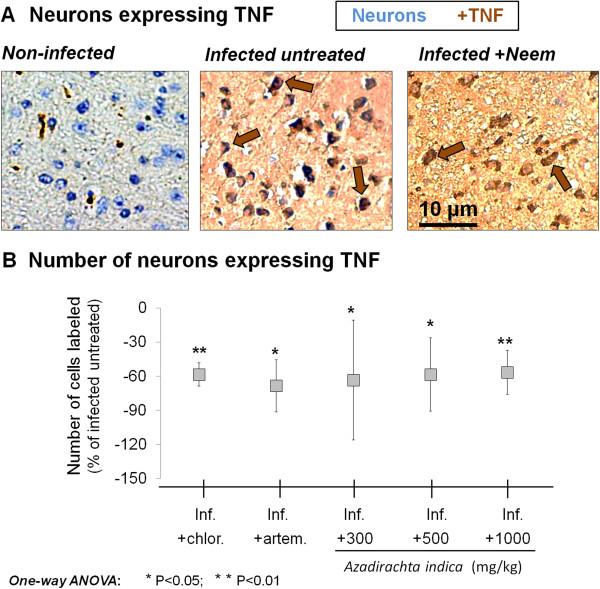
**TNF expression. A**. Micrographs showing the expression of tumor necrosis factor (TNF) in pyramidal neurons of representative examples of (from left): an uninfected mouse, an untreated Plasmodium berghei-infected mouse (inf.), and a P. berghei-infected mouse treated with Azadirachta indica crude extract. **B**. Effect of **A**. indica on the expression of TNF in pyramidal neurons of P. berghei-infected mice. Note the significant decrease in expression following treatment with chloroquine (chlor.), artemether (artem.), or any of the doses of **A**. indica extract used.

The effect of *A. indica* on TNF expression in pyramidal neurons of *P. berghei*-infected mice is shown in Figure 4. Pyramidal neurons of mice treated with *A. indica* extract displayed a significant decrease in TNF expression at all doses used, in comparison to the infected untreated group (P < 0.01 at 1000 mg/kg). This effect was comparable to the decrease in TNF expression induced by chloroquine (P < 0.01 *vs*. infected untreated group) or artemether (P < 0.05 *vs*. infected untreated group).

### Extract effect on NOS expression

**Figure 5:**
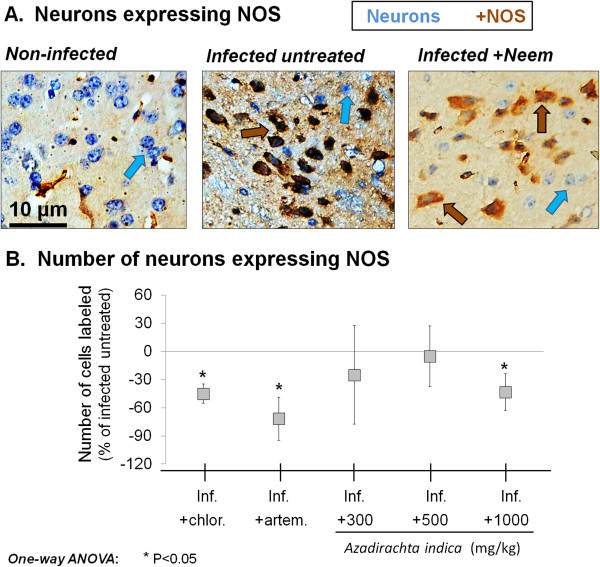
**NOS expression. A**. Micrographs showing the expression of nitric oxide synthase (NOS) in pyramidal neurons of representative examples of (from left): an uninfected mouse, an untreated Plasmodium berghei-infected mouse (inf.), and a P. berghei-infected mouse treated with Azadirachta indica crude extract. **B**. Effect of **A**. indica on the expression of NOS in pyramidal neurons of P. berghei-infected mice. Note the significant decrease in expression following treatment with chloroquine, artemether, and **A**. indica extract highest dose. Data are mean ± SEM.

The effect of *A. indica* on the expression of NOS in *P. berghei*-infected mice pyramidal neuron is shown in Figure 5. *Azadirachta indica* extract induced a significant decrease in NOS expression, in comparison to the infected untreated group. However, such decrease was statistically significant and comparable to the effect of chloroquine (P < 0.05 *vs*. infected untreated group) or artemether (P < 0.05 *vs*. infected untreated group) only at the highest dose used (1000 mg/kg, P < 0.05).

### Extract effect on brain oedema severity

**Figure 6:**
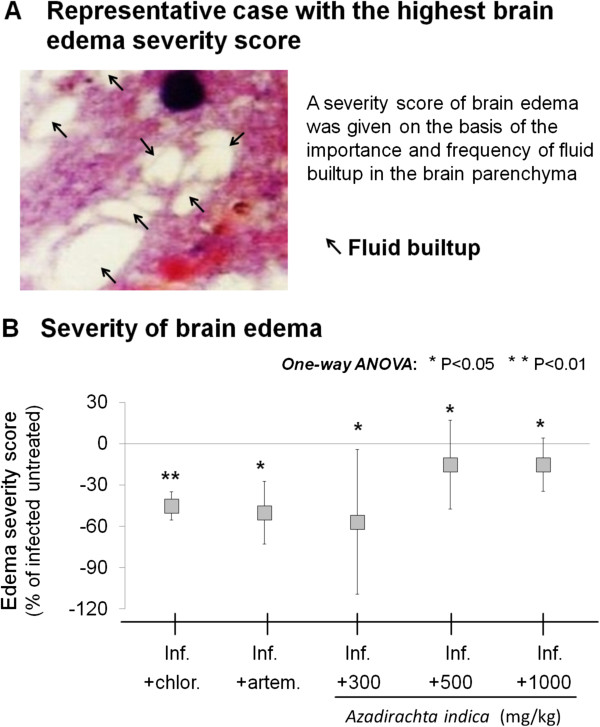
**Brain oedema. A**. Micrograph showing a representative case of highest brain oedema severity score. **B**. Effect of Azadirachta indica on the severity of brain oedema in Plasmodium berghei-infected (inf.) mice. Note the significant decrease in brain oedema severity following treatment with chloroquine (chlor.), artemether (artem.), or any of the doses of **A**. indica extract used. Data are mean ± SEM.

The effect of *A. indica* extract on brain oedema severity in *P. berghei*-infected mice is shown in Figure 6. *Azadirachta indica* extract induced a significant decrease in brain oedema severity at all doses used, in comparison to the infected untreated group (P < 0.05), which was comparable to the effect of chloroquine (P < 0.01 *vs*. infected untreated group) or artemether (P < 0.05 *vs*. infected untreated group).

## Discussion

The present study points out the protective effects of *A. indica* extract on pyramidal neurons in experimental cerebral malaria induced by *P. berghei*-infection in mice.

### Protective effects on pyramidal neurons

Pyramidal neuron density study revealed comparable values between *P. berghei*-infected mice treated with the extract of *A. indica* and uninfected mice, which were significantly different from untreated *P. berghei*-infected mice (disease control group) where a significant decrease in pyramidal neuron density, characteristic of cerebral malaria insult [14,18-20], was observed. These findings suggest that the extract protected pyramidal neurons from cerebral malaria-induced apoptosis. In order to further characterize these effects, the expression of apoptosis markers was studied, considering the importance of this phenomenon in cerebral malaria-associated encephalopathy [7-9].

### Striking decrease in the expression of apoptosis markers

The markers of apoptosis studied were caspase 3, Fas, and Fas ligand, which are commonly used for the detection of apoptosis in neuronal populations [13,19]. *Azadirachta indica* extract induced a striking decrease in caspase 3, Fas, and Fas ligand expressions on pyramidal neurons of *P. berghei*-infected mice, particularly at the highest dose used. Considering that these markers are associated with the risk for neuron apoptosis [13,19], the present finding indicates that *A. indica* extract has anti-apoptotic effects on pyramidal neurons in experimental cerebral malaria. The latter effects probably account for at least part of the protective effects of the extract against pyramidal neuron death in treated *P. berghei*-infected mice. Considering that the pro-inflammatory environment, and particularly infiltrating immune cells, contribute to the detrimental effects of cerebral malaria on neurons, including apoptosis in sensible neuronal populations [12,14,42], through extrinsic mediators of apoptosis such as TNF and NOS [12,43,44], we have addressed the effect of *A. indica* extract on these inflammatory triggers of apoptosis in *P. berghei*-infected mice.

### Mitigation of neuroinflammatory triggers of apoptosis and brain oedema severity

Treatment with *A. indica* extract induced decreases in pyramidal neuron expression of TNF and NOS in *P. berghei*-infected mice as compared with disease control group, suggesting that the extract modulated inflammation in the brain parenchyma. Besides, considering that brain oedema is a major causative of the inflammatory response in the brain parenchyma (through infiltrating danger or pathogen-associated molecules [10,11]), the decrease in oedema severity observed in groups treated with the extract in the present study, indicate that the aforementioned anti-inflammatory effects may be mediated, at least in part, by protective effects on brain vessels. These findings are in agreement with our precedent observations in Purkinje cells of *P. berghei-*infected mice, where *A. indica* also displayed neuroprotective effects [16]. Comparable effects of *A. indica* extract have been reported in other models of detrimental neuroinflammation associated with brain oedema, including cerebral post-ischemic reperfusion and hypoperfusion in rats [45,46]. Thus, the findings herein reported confirm that *A. indica* extract has neuroprotective effects in experimental cerebral malaria, and indicate that such effect may be mediated through protective effects on brain vessels and the consequent decrease in neuroinflammation severity. Not surprisingly and interestingly, chloroquine and artemether, drugs in use for cerebral malaria treatment in the field [37,47], also induced similar effects, corroborating their beneficial effects reported in humans, and pointing out at least part of the mechanisms accounting for these effects. It appears, therefore, that future studies aiming at unraveling the active principle(s) accounting for the beneficial effects of *A. indica* extract reported may provide key elements for the development of new drugs for cerebral malaria. Subjecting the bioactive agents of Azadirachta indica which have already been characterized, such as gedunin [33], [48], to a similar study may provide better insights into the neuroprotective effects herein described.

## Conclusions

*Azadirachta indica* extract did not protected infected mice from death from cerebral malaria, although the highest doses slightly delayed death time. However, *A. indica* extract displayed neuroprotective effects mediated probably by mitigating oedema build up, and modulating both neuroinflammatory triggers of apoptosis and apoptosis signaling pathways. The use of extracts of *A. indica* in African and Asian folk medicine and practices for the treatment of malaria and cerebral malaria is justified. Characterization of the active principle(s) accounting for these effects will probably provide new drugs, which are particularly needed in the current context of increased chemoresistance to chloroquine.

## Competing interests

The authors declare that they have no competing interests.

## Authors’ contributions

MF, EAK, SAK, MAA, AM, PFSE, and YHA participated in the design of the experiment. SB and MF performed the experiments. MF, EAK, SAK, MAA, AM, SB, YHA and PFSE participated in the preparation of the manuscript. MF and PFSE analysed the data, prepared the figures, and were in charge of the manuscript submission. All authors read and approved the final manuscript.
